# Assessing Technical Feasibility and Acceptability of Telehealth Palliative Care in Nursing Homes

**DOI:** 10.1089/pmr.2022.0002

**Published:** 2022-08-22

**Authors:** Caroline E. Stephens, Theresa A. Allison, Lynn A. Flint, Daniel David, Victoria Wertz, Elizabeth Halifax, Pamela Barrientos, Christine S. Ritchie

**Affiliations:** ^1^ Division of Health Systems and Community Based Care, University of Utah College of Nursing, Salt Lake City, Utah, USA.; ^2^ Division of Geriatrics, University of California, San Francisco, San Francisco, California, USA.; ^3^ NYU Rory Meyers College of Nursing, New York City, New York, USA.; ^4^ Infusion Treatment Area, Stanford Health Care, Stanford, California, USA.; ^5^ Department of Social and Behavioral Sciences, University of California, San Francisco, San Francisco, California, USA.; ^6^ Mongan Institute Center for Aging and Serious Illness, Massachusetts General Hospital, Boston, Massachusetts, USA.

**Keywords:** acceptability, feasibility, nursing home, palliative care, telehealth

## Abstract

**Background:**

Over two-thirds of nursing home (NH) residents are eligible for palliative care (PC), yet few receive it, particularly outside of hospice. Little is known about the technical feasibility and acceptability of using telehealth for PC consultations in NHs.

**Objective:**

To determine the technical feasibility and acceptability of PC telehealth for NH residents seen by a PC team in the hospital in the previous 30 days.

**Design:**

Mixed methods study including data collection from field observations, focus groups about the telehealth experience with content analysis, and a web-based survey about technical feasibility and acceptability.

**Sample and Approach:**

Eighteen participants (six PC-eligible NH residents, one PC physician, five family members, six NH nurses) were recruited in 2016 to participate in one of six PC video visits followed by a video-based focus group and web-based survey.

**Results:**

All participants were comfortable with the PC video visit format, believed it could improve communication and care coordination, and reported they could see themselves using telehealth in the near future. For technical feasibility, audio quality was rated mostly good/very good (71%) and visual quality was rated fair (50%).

**Conclusions:**

PC video visits are technically feasible and acceptable to NH residents, families, and staff, representing an innovative and relatively low-cost opportunity to improve access to needed NH-based PC services. Assessing stakeholder perspectives on the use of this technology can help inform the selection of the proper telehealth platform to meet the clinical and infrastructure needs, as well as protocol modifications required before testing in a larger trial.

## Introduction

Over two-thirds of nursing home (NH) residents are eligible for palliative care (PC), yet few receive it.^1^ PC benefits residents and families by enhancing symptom management, improving satisfaction and care quality, and reducing burdensome hospital transfers, especially at the end of life (EOL).^2^ Because specialty PC is not a common part of NH care and few specialists are available,^3–5^ alternative care solutions are needed.

Digital advances in health care have demonstrated potential to enhance health outcomes, particularly for hard-to-reach populations.^6,7^ Telehealth, or the remote provision of care for patients through video communication technologies, may make PC consultation a viable option for NH residents with serious illnesses.^8^ Telehealth has been shown to optimize NH care through access to after-hours consultation and to specialty consultations (psychiatry, neurology, wound care, etc.); however, little is known about the technical feasibility and acceptability of using telehealth to improve access to PC in this setting.^9^ Successful adoption and implementation of sustainable telehealth systems necessitate stakeholder support and identification of the factors that influence successful adoption.

## Design and Procedure

As a formative step in determining technical feasibility and acceptability of NH-based PC telehealth, we conducted an integrated mixed-methods multistakeholder technical feasibility pilot study between March and December 2016. Our approach included participant observation with fieldnotes, PC video visit focus group discussions using VSee,^10^ and a web-based survey.

To develop a multistakeholder perspective on the PC telehealth consult and platform itself, we recruited a purposive sample of NH residents, their family members, and staff from three California community-based NHs. Study NHs were all new to telehealth, except for use of asynchronous telehealth wound care and rare neurology follow-up for select Health Maintenance Organization (HMO) patients. They were also part of a larger readmission reduction collaborative that found that poor communication and care coordination, as well as unmet PC needs, were common drivers of NH hospital transfers.^1,2,11^

A hospital-based PC physician was recruited as study participant and clinician to conduct the PC visit. Such an approach fostered a standardized process for conducting each of the six PC visits, while allowing us to iteratively collect data and make observations about technical feasibility and interactions between and among the different stakeholders. Eligible NH residents were English speaking, not acutely ill, and had been seen in a hospital by a PC team within the past 30 days. Family members of eligible residents were included if they were English speaking and identified as the closest relative or next of kin. Eligible NH staff included licensed nurses who recently cared for and/or had knowledge of the enrolled resident. Informed assent or consent was obtained from all participants.

Multiperson video visits were initiated with the assistance of research nurses colocated with participants at the NH (resident, nurse, and sometimes family), hospital (PC physician), and off-site (family). The PC physician conducted each of the PC visits that included conversations about symptoms and goals of care, as well as screen sharing of the Physician's Orders for Life Sustaining Treatment (POLST) form.

After each 20-minute PC video visit, the principal investigator led a video-based focus group discussion with participants about their experience using a semistructured interview guide (Supplementary Appendix A1). Following the focus group, participants used a study tablet or laptop to complete a web-based survey about the acceptability and feasibility of the visit. Research nurses assisted in survey completion as needed.

During and after each video visit, research nurses recorded field notes of the experience, noting technical challenges and participants' verbal and nonverbal responses to, and interactions with, the visit. Within 24 hours of each visit, all research team members convened for 30-minute debriefing sessions to discuss experiences and findings, troubleshoot technology issues, and iteratively adapt and refine study protocols. Content analysis^12,13^ was performed on field and group discussion notes, and validated against the debriefing sessions to provide a more nuanced understanding of feasibility and acceptability. This study was approved by the university's institutional review board.

## Results

Eighteen individuals participated in the study (six NH residents, one PC physician, five family members, six NH nurses) (Table 1). Nearly three-quarters of participants reported previous use of a technology device for a video visit with another person within the past 5 years, but none had experience with health care-related video visits. Five NH resident participants included a family member or friend in the video visit; one had no identifiable family, friend, or surrogate decision maker. One resident was unexpectedly taken to dialysis at the time of his scheduled video appointment, so the first part of the visit was conducted at the dialysis center and concluded at the NH upon return.

**Table 1.** Characteristics of Palliative Care Video Visit Participants (*n* = 18)

Variable	***n*** (%)
Age (years) (%)	
31–40	4 (22)
41–50	3 (17)
51–60	1 (6)
61–70	4 (22)
80+	6 (33)
Race/ethnicity (%)	
Hispanic/Latino	0 (0)
Black/African American	3 (17)
Asian	7 (39)
White	4 (22)
Unknown/not reported	4 (22)
Prior experience with devices (%)	
Smartphone	10 (56)
Tablet	11 (61)
Laptop	16 (89)
Desktop	13 (72)
Prior experience with video communication (e.g., Skype, FaceTime) (%)	
Any	13 (72)
Current	10 (77)
<1 year ago	2 (15)
1–5 years ago	1 (8)

Observations and focus groups revealed overall acceptability and appreciation across stakeholders. In particular, family members believed the video visit allowed for more interaction with health care providers in general and allowed for clarification of certain issues regarding resident care. Off-site family members appreciated being able to view and talk with their loved ones from a distance, as they were able to gain a better sense of how their loved ones were doing emotionally and physically. One family member reported the value of seing her loved one's “personality on the screen.” One sister described the powerful impact of seeing her brother undergoing dialysis and the toll it took on him.

Strikingly, the only resident who reported no prior technology experience was eager to participate in the video visit and described having a meaningful conversation, including review of his POLST. Although the primary goal of this study was to understand participants' experiences using technology, participants also described new insights attributed to topics discussed, particularly goals of care. For example, the PC physician reported initial skepticism about NH PC telehealth, but, by the end of the study, described telehealth as having the potential to be useful, particularly using the screen-share function to “control” presentation of POLST information to systematically review and discuss.

One son described reaching emotional acceptance of his mother's imminent death after the PC physician explained that people at the EOL often stop eating. Such guidance had not been otherwise available. He said the video visit discussion allowed him “…to move on, when previously I was in denial.”

Several feasibility issues were identified. Poor Internet connectivity during initial tests led to screen freeze, loss of synchronicity, and poor volume. Environmental interference (change in resident dialysis schedule for planned video visit, noisy environments, staff accidentally walking into the video visit, etc.), as well as resident and/or family auditory, visual, and mild aphasia challenges created additional barriers. Solutions evolved iteratively, including the addition of amplification devices, adaptive large print communication tools, mobile hot spot devices, and trial of alternative platforms. Despite these challenges, 71% rated audio quality as good or very good and 50% rated visual quality as fair (Table 2).

**Table 2.** Acceptability and Feasibility of Palliative Care Video Visit (*n* = 14)

Variable	***n*** (%)
Comfortable with palliative care video visit (%)	
Strongly agree	7 (50)
Agree	7 (50)
Neutral	0 (0)
Disagree	0 (0)
Strongly disagree	0 (0)
Would use video visit technology in near future (%)	
Yes	14 (100)
For own care?	12 (86)
For family/loved one's care?	13 (93)
For patients?	11 (79)
In place of work?	12 (86)
Would improve communication between family members and among those providing care (%)	
Strongly agree	11 (79)
Agree	3 (21)
Neutral	0 (0)
Disagree	0 (0)
Strongly disagree	0 (0)
Would improve coordination of care between nursing homes and hospitals (%)	
Strongly agree	9 (64)
Agree	5 (36)
Neutral	0 (0)
Disagree	0 (0)
Strongly disagree	0 (0)
Quality of communication with the palliative care video visit group (%)	
Very poor	0 (0)
Poor	0 (0)
Fair	3 (21)
Good	5 (36)
Very good	6 (43)
Audio quality (%)	
Very poor	0 (0)
Poor	0 (0)
Fair	4 (29)
Good	8 (57)
Very good	2 (14)
Visual quality (%)	
Very poor	1 (7)
Poor	0 (0)
Fair	6 (43)
Good	5 (36)
Very good	2 (14)

Missing complete REDCap survey data for four residents.

The survey data triangulated and validated qualitative findings. Participants were comfortable with the video visit format and reported they could see themselves using telehealth in the near future (Table 2). Participants further agreed/strongly agreed that this technology could improve communication between residents, families, and providers and improve coordination of care between NHs and hospitals.

## Conclusions

Before the COVID-19 pandemic, we found PC video visits to be technically feasible and acceptable to NH residents, families, and staff, representing an innovative opportunity to improve access to needed NH-based PC services. Such virtual visits were perceived as very beneficial, having the potential to improve communication between stakeholders and care coordination. They allowed for greater engagement of family at a distance; a visual understanding and appreciation of resident status; discussion and clarification of goals, values, and preferences; and flexibility in adapting to the NH environment/situation.

These study findings build on recent telehealth PC and other subspecialty feasiblity studies conducted in Canadian long-term care homes^14^ and VA-contracted community nursing facilities^15^ underscoring the feasibility and acceptability of videoconferencing as a means of PC provision. The COVID-19 pandemic and ongoing changes in Centers for Medicare & Medicaid Services (CMS) telehealth guidelines^16^ have obscured underlying preferences because of infection control imperatives to increase telehealth encounters.^17^ This study provides evidence of the technical feasibility when the system is not in crisis.

Of note, this was a small pilot study conducted at three NHs in California and is, therefore, limited in generalizability. Although incomplete survey data from four of the six residents limited input from the resident perspective, it provided useful information regarding participant eligibility, timing, and length of visits and surveys. Another potential limitation relates to recruitment of residents who received a hospital PC consult and/or may have had a predisposing interest in technology-assisted PC services. Nevertheless, there was a uniformly positive response to using telehealth for PC in the NH setting, providing a strong indication that such visits would be welcomed by all stakeholders.

In technologically naive environments, such as sites in this study and most U.S. NHs in general, the assessment of feasibility and accessibility of video visits is a critical initial step in the development of video-based PC consultation. Although establishing such technical feasibility is a fairly straightforward first step in any such effort to digitally transform health care, it is also important to consider acceptability concerns.^7^ Some may fear that the increasing use of technology in health and care settings may come at the expense of human connection (i.e., high tech—low touch).^7^

Given that overall telehealth use in NHs significantly expanded during the COVID-19 pandemic and it is expected to continue,^18^ it will be essential to maintain a discerning approach to the use of such digital health technologies to ensure they are both high tech *and* high touch.^7^ These pilot data show clear acceptability to residents, families, and the interdisciplinary team. Developing an understanding of factors that influence successful adoption will be key to the implementation of sustainable telehealth systems. Only when the artificial divide between high tech and high-touch care is bridged will PC be able to meet the changing needs and expectations of consumers.^7^

### Next steps

A comprehensive pilot study is in progress that builds on this research with aims to further refine and optimize care processes and protocols postpandemic—critical components in maximizing acceptability, replicability, and dissemination of telehealth interventions, particularly among PC-eligible NH populations.

## Supplementary Material

Supplemental data

## Supplementary Material

Supplementary Appendix A1

## Abbreviations Used

EOL: end of life
NH: nursing home
PC: palliative care
POLST: Physician's Orders for Life Sustaining Treatment
