# Supplementary material for: Assessing Technical Feasibility and Acceptability of Telehealth Palliative Care in Nursing Homes
Source: Palliat Med Rep. 2022 Aug 22;3(1):181–5. doi: 10.1089/pmr.2022.0002 (PMC9438441; doi:10.1089/pmr.2022.0002)
Supplement: Supplemental data [file Suppl_AppA1.docx]

**APPENDIX A**

**Technical Feasibility Pilot Video Visit Interview Guide**

**Overview of Procedure**

The goal is to start the video visit no more than 10 minutes after appointment starts. During this time:

- Thank you for coming today/introductions
- Obtain informed consent as necessary (if not done prior to meeting that day)
- Familiarize participants with software (demonstrate with test call and show chat options)
- Familiarize participants with the expectations we have about how the video visit conversation will be conducted, and their role. Specifically:

We are asking you all to discuss (residents name’s) general well-being since their hospital stay in order to test how this technology can be used to communicate. Underscore, that this will be a conversation, not a clinical visit.

- Before we begin, do you have any questions?

**Interview Guide**

1. Introduction

Thank you all for coming today! We appreciate you taking time out of your busy schedules to participate in this video visit.

We are currently talking to each other from <insert #> locations, and I would like to introduce members of the research team and others participating today (introductions).

This video visit is the first part of a pilot study looking at how we might improve access to palliative care for people in nursing homes.

The goals of this and the other initial video visits are to:

- Figure out the best way to use this technology
- Find out what you thought about the experience of communicating in this way

Your participation and experience is invaluable in helping us to achieve these goals!

Here is the plan for our visit:

- We will go over ground rules
- [Name of PC provider] will ask you about your general wellbeing since your hospital stay to test how this technology can be used to communicate. This will be a conversation, not a clinical visit.
- When the video visit is complete, we will ask you as a group about the experience of having this discussion using this technology. We want to know what you liked about it, what you did not like about it, and what you think we could do differently.
- Before leaving, please answer the questions sent to you on the device you were using

2. Ground Rules

**What takes place during this test remains confidential**

- Although this is not a clinical visit, these discussions are covered by the same rules of confidentiality that all medical records have.
- We want you to feel safe sharing all information.

**There are no right or wrong answers**

- Every person’s experiences and opinions are important

**We will be audio recording the discussions**

- We want to capture everything you have to say about this important topic
- We don’t identify anyone by name in our report. You will remain anonymous.

**Please feel free to ask questions at any time** during or after the video visit

**You can at any time ask to stop taking part in the video visit**, without any consequences.

3. Conversation led by PC Provider

We would now like (names of PC provider, NH nurse, resident and family member) to have a conversation about (resident’s) general wellbeing since their hospital stay. This is to test how this technology can be used to communicate.

4. Semi–Structured Group Interview

Now I would like to hear what you thought about the video visit technology we used today.

**How was your experience using the video visit technology?**

*Probes:*

- How did it feel to be able to communicate with the other members of the group using this technology?
- Did you have any challenges setting up and/or connecting with the other individuals on the videoconferencing call?
- Can you describe them?

**How would you describe the strengths and weaknesses of this technology?**

*Probes:*

- How was the technology helpful?
- Can you tell me more about that?
- Did you experience any problems using this technology?
  - Can you describe them?
- How do you suggest we could have done things differently?
- How do you feel about issues of privacy and security when using this technology?

Prompts for use during interview:

- Can you tell me more about that?
- What do you mean by that?
- What did that mean to you?

*Thank you so much for your time and sharing your thoughts. Is there anything else you would like to say about your experience or what we talked about, or that you think I should know?*

**5.** In Conclusion

We will be signing off now. Before you leave, the research team member who is with you will:

- Help you complete the brief online survey.
- Give you your gift card (or arrange delivery of your gift card) to thank you for sharing your time and expertise.
